# Seroprevalence and associated risk factors of Dengue fever in Kassala state, eastern Sudan

**DOI:** 10.1371/journal.pntd.0008918

**Published:** 2020-12-09

**Authors:** Arwa Elaagip, Khider Alsedig, Omnia Altahir, Tellal Ageep, Ayman Ahmed, Hanaa Adli Siam, Abdallah M. Samy, Waleed Mohamed, Fatima Khalid, Suhaib Gumaa, Leonard Mboera, Calvin Sindato, Linzy Elton, Alimuddin Zumla, Najmul Haider, Richard Kock, Muzamil Mahdi Abdel Hamid

**Affiliations:** 1 Department of Parasitology and Medical Entomology, Faculty of Medical Laboratory Sciences, University of Khartoum, Khartoum, Sudan; 2 Department of Parasitology and Medical Entomology, Institute of Endemic Diseases, University of Khartoum, Khartoum, Sudan; 3 Department of Medical Entomology, National Public Health Laboratory, Federal Ministry of Health, Khartoum, Sudan; 4 Department of Epidemiology, Tropical Medicine Research Institute, National Center for Research, Khartoum, Sudan; 5 Entomology Department, Faculty of Science, Ain Shams University, Abbassia, Cairo, Egypt; 6 Department of Biochemistry, Faculty of Medicine and Health Sciences, University of Kassala, Kassala, Sudan; 7 Department of Immunology and Biotechnology, Tropical Medicine Research Institute, National Center for Research, Khartoum, Sudan; 8 SACIDS Foundation for One Health, Sokoine University of Agriculture, Morogoro, Tanzania; 9 National Institute for Medical Research, Tabora, Tanzania; 10 Centre for Clinical Microbiology, Department of Infection, Division of Infection and Immunity, Royal Free Campus, University College London, London, United Kingdom; 11 Royal Veterinary College (RVC), London, United Kingdom; Australian Red Cross Lifelood, AUSTRALIA

## Abstract

Dengue is a rapidly growing public health threat in Kassala state, eastern Sudan. The objective of this study was to determine the seroprevalence, entomological transmission indices, and socioeconomic risk factors associated with dengue in this region. A cross-sectional community-based study was conducted in four dengue-endemic sites; Khatmia, West Gash, Thoriba, and Shokriya between March 2016 to March 2017. Enzyme-linked immunosorbent assay (ELISA) of immunoglobulin G (IgG) was used to determine the prevalence of dengue virus among the study participants. An entomological survey was conducted using pyrethrum spray catch and dipping for the collection of adults and aquatic stages of *Aedes aegypti*, respectively. Ribonucleic acid was extracted from the buffy coat of participants as well as from adult female *Ae*. *aegypti* to assess the possible circulation of dengue virus using Reverse Transcription Polymerase Chain Reaction (RT-PCR). Multiple logistic regression model was used to estimate the association between potential risk factors and dengue seropositivity. A total of 409 persons were recruited to the study: 45.5% were in the 20–39 years’ age category; 57.9% were living in houses with 6–10 persons; and 29.1% had at most secondary school education. In the majority (65.8%) of the households, the socioeconomic status was low (P<0.001). Long-lasting insecticide-treated bed nets were used in 56.5% of the households. Over three-quarters (77.8%) claimed not to have experienced febrile illness in the last three months. Routine entomological survey across Kassala state identified a total of 3,304 larvae and 390 pupae *Ae*. *aegypti*, respectively. The overall house index was 32.8% and Breteau Index was 35.96% (146/406). The overall pupal demographic index was 13.31%, and the pupal children index was 97.26%. Antibodies against IgG were detected from 66 (42.04%) out of a total of 157 sera. Twenty-two positive sera (75.9%) were collected from Khatmia. A total of 329 adults *Ae*. *aegypti* were identified but only one (0.3%) was positive for DENV in Khatmia. Finally, four independent risk factors were identified to derive dengue circulation in Kassala: elder age (> 60 years) (OR 6.31, CI 1.09–36.36); type of bathroom (OR 3.52, CI 1.35–9.20); using water-based air conditioner (OR 6.90, CI 1.78–26.85) and previous infection of any household member with dengue (OR 28.73, CI 3.31–249.63). Our findings suggest that Kassala state is facing an increasing occurrence of dengue and emphasizes the need for developing appropriate interventions to address the identified risk factors, and place control programs into actions. Establishment of routine dengue epidemiological and entomological surveillance, and climate warning systems will contribute to early warning and timely detection and response to emerging outbreaks.

## Author summary

Dengue is a rapidly growing public health threat in Sudan. Kassala state is facing a major outbreak of the mosquito-borne dengue virus. This recent outbreak alarmed the local health authorities to establish a successful control program. However, lack of data obstructs their roles to achieve this goal. Here, we provided a detailed picture on the seroprevalence of dengue virus, entomological indices, and natural mosquito infection across Kassala state, Sudan. The study also identified key factors associated with the recent dengue outbreaks in Sudan. All these findings marked the importance to establish successful routine vector and dengue surveillance. These active surveillances should consider sensitive early warning systems providing early anticipation and timely detection and response to the future outbreaks in Sudan.

## Introduction

Dengue virus (DENV) is one of the increasingly emerging arthropod-borne diseases worldwide. It is transmitted by the bites of mosquitoes of the genus *Aedes*; *Aedes aegypti* has been recognized as the primary dengue vector [1,2] while *Aedes albopictus* has been implicated as a DENV vector in diverse outbreaks across several endemic countries [3]. Cases of DENV are widely distributed in tropical and sub-tropical countries, mostly in urban and semi-urban settings [3]. The global incidence of dengue has grown dramatically in recent decades [4]. A recent study estimated half of the world's population in dengue risk [5]. It is estimated that about 100–400 million new infections of DENV occur annually around the globe [4,6,7].

Dengue virus infections can be caused by one of the four genetically related serotypes (DENV-1–4) that circulate in several parts of the world [8]. Dengue virus is a non-segmented, positive single-stranded, enveloped RNA virus, belonging to the genus *Flavivirus* of the family *Flaviviridae* [9]. The mosquito vector *Ae*. *aegypti* is domesticated and breeds in small bodies of water, including various containers found around houses in urban areas of the tropical and subtropical world [10]. It feeds exclusively on humans during the daytime and rests indoors [10,11].

Dengue infection has different clinical presentations ranging from a self-limiting flu-like illness to fatal severe forms of dengue haemorrhagic fever and dengue shock syndrome [12]. The majority of DENV infections are asymptomatic, and the febrile illness is characterized by headache, rash, muscle pain, and other symptoms [13,14]. It is difficult to clinically distinguish between dengue and other arboviral infections without laboratory diagnosis to confirm virus circulation [15,16,17]. However, the early diagnosis seems to have a significant positive impact on patient recovery following appropriate case management.

Dengue has a negative linear economic impact as the global incidence of dengue has grown dramatically with the spread of several complex factors associated with its re-emergence events [18]. These complex factors are associated with rapid human population growth, unplanned and uncontrolled urbanization, and increased international travel [19,20,21,22]. Recently, epidemics of DENV have increased in African countries, mostly in the eastern Africa [16]. The emergence of DENV in the Darfur area of the western Sudan was associated with the post-conflict social and environmental change [23]. It is a challenge to understand the epidemiology of DENV in the African settings for multiple reasons, such as inadequate surveillance, under-reporting of illness syndromes, and poor diagnostic capacity. These situations delay outbreaks recognition and discovery [16,24].

In Sudan, dengue cases were identified for the first time in 1906 from patients at Port Sudan city and Swakin port [25]. Dengue cases have been reported from 12 Sudanese states between 1984 and 2015 [26]. Most dengue cases were diagnosed in Port Sudan and Kassala states in eastern Sudan [16,26]. DENV-1 and DENV-2 were recognized as the most common serotypes of dengue virus in all outbreaks in Sudan [16,27]. Kassala state has experienced an increased incidence of dengue outbreaks during the last years [28,29]. The state is compounded by the huge population, poor economic situation, cross-border movement, poor medical and diagnostic facilities, inadequate mosquito control, and all the ground conditions that favour the expansion of the vector breeding sites [29]. Active surveillance of human cases and mosquito vectors in vulnerable dengue populations can help to monitor the epidemiological picture of local human and mosquito populations harbouring specific DENV serotypes and provides an early warning data to anticipate impending epidemics.

Here, we report the findings of study carried out to assess seroprevalence of dengue virus, entomological transmission indices, and socioeconomic risk factors associated with recent dengue circulation in Kassala state, eastern Sudan.

## Methods

### Ethics Statement

All study participants received detailed information regarding the study procedures and objectives using their local language. Each participant agreed verbally and signed the informed consent to participate in this study. The guardian of all minors agreed and signed the consent on their behalf. Ethical approval for the study was provided by the Faculty of Medicine, University of Kassala, Sudan.

### Study area

**Fig 1:**
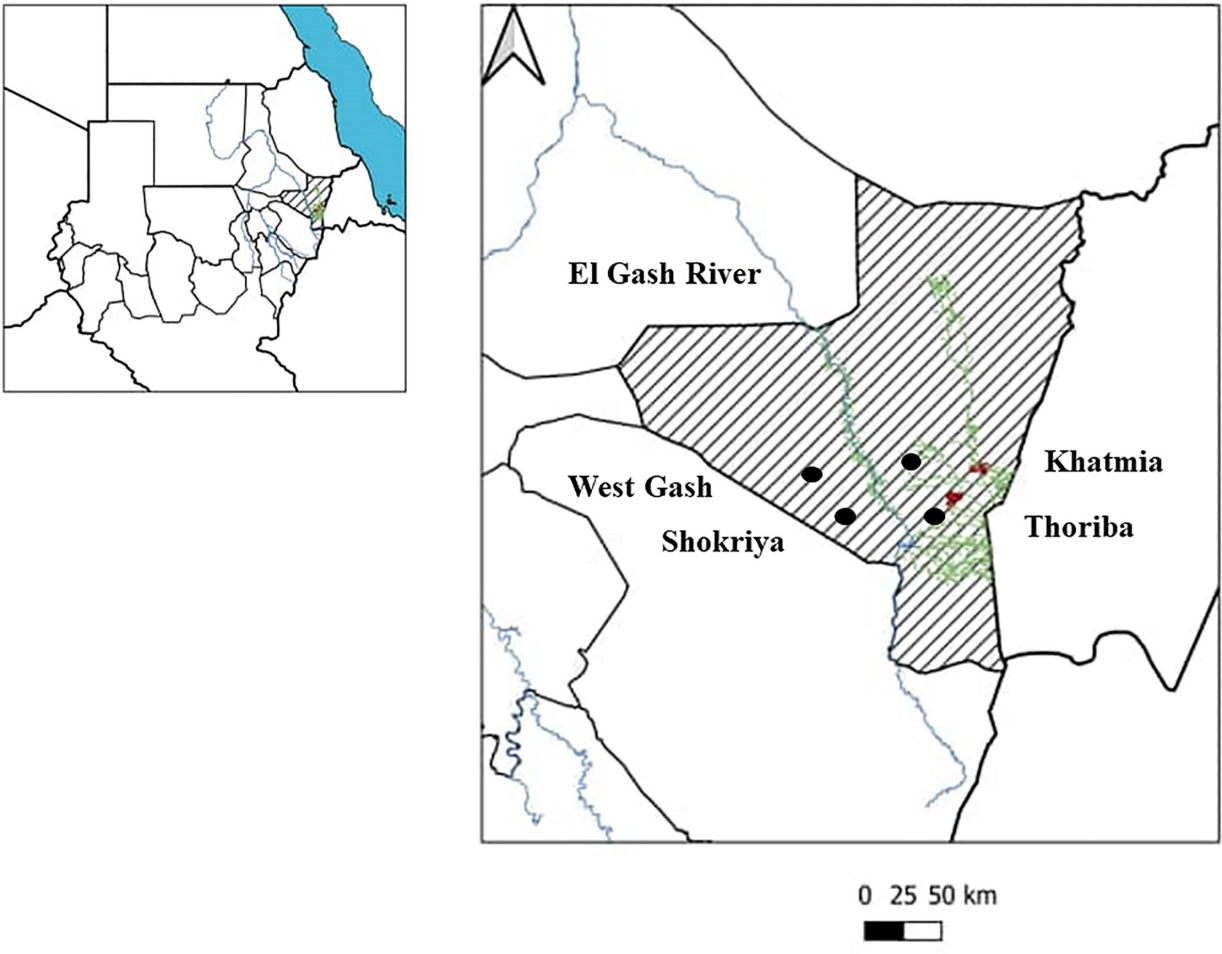
Regional and local map of the study sites in Kassala state, eastern Sudan. The areas highlighted with black circles represent the study sites in Khatmia, Thoriba, West Gash, and Shokriya.

Kassala state is located in eastern Sudan (10°12′N 34°19′E) (Fig 1). Eritrea and Ethiopia border it to the east, Red Sea state to the north, the states of Khartoum and River Nile to the west, and Gedarif state to the southwest. Kassala state land space is 42.282 km^2^ with 401,477 total number of households according to World Population Review, 2019 (https://worldpopulationreview.com/countries/sudan-population/). In the northern parts of the state, the climate is coastal which features high humidity and extreme variation in the seasonal temperature, while in the other parts; the environment is desert, semi-desert, and savannah habitats. The climate in Kassala is associated with large irrigated fruit farms. The annual rainfall rate in Kassala state ranges from 350 to 911 mm; however, the mean daily temperature ranges from 17°C to 42°C. The average family size is 6 individuals/family. The sources for drinking water include superficial, ground, and deep groundwater [30]. The percentage of the population with access to clean drinking water is 68%, and 40% in the urban and rural areas, respectively [30].

### Study design and sampling technique

A cross-sectional community-based study was conducted from March 2016 to March 2017. A multi-stage sampling approach was used to select clusters (administrative units) then selection of households using a random sampling technique. Within the household, residents aged more than five years old were randomly selected for interview and blood collection [30,31]. Additionally, entomological survey was conducted in each selected household. According to the sampling approach, four clusters were chosen for the study (Khatmia, West Gash, Thoriba, and Shokriya) in light of their higher dengue incidence. The four localities were the most affected areas by dengue infections among the recent outbreaks in Kassala state [Kassala Ministry of Health, personal communication]. The sample size was calculated based on the estimated prevalence of dengue in Kassala state [30,31].

The household survey was conducted during the wet season (May–October) and dry season (November–March). All the selected households were georeferenced using a geographical positioning system (GPS) hand-held device (Garmin Ltd., Kansas, USA). Microclimatic data (e.g., temperature and relative humidity) were recorded within all selected households.

### ELISA IgG analysis

IgG antibodies specific for dengue serotypes (1, 2, 3, and 4) were detected using Dengue IgG Capture enzyme-linked immunosorbent assay (ELISA) kit (Panbio, Inverness Medical Innovations, Australia) [32] following the manufacturer instructions.

### Entomological investigations

In the larval and pupal survey, all water containers located indoors and outdoors in the selected houses were examined for aquatic stages of dengue vectors. Containers were recorded according to the type, the source of water, the location, the presence of a fitting lid, and the presence of aquatic stages.

Adult *Aedes* mosquitoes were collected from rooms using pyrethrum spray catch (PSC) [33]. Adult samples were preserved individually in specimen tubes in RNAlater (Ambion) and transported to the laboratory for morphological identification and viral dengue RNA detection using reverse transcriptase RT- PCR [27]. Morphological identification of collected adults and aquatic stages of mosquitoes was done using the taxonomy key of Huang [34].

### Sample processing, RNA extraction, and Reverse transcriptase PCR

One individual (age > 5 years) in each enrolled house was asked to provide a 2 ml venous blood sample for serology and RNA extraction. Blood was collected in EDTA tubes. Plasma was separated by centrifugation at 1,500 rpm for 10 minutes at room temperature. The buffy coat was transferred into separate tubes and stored at -20°C until later use. The RNA was extracted from the buffy coat [35] as well as from adult female *Ae*. *aegypti* using Easy-blue total RNA extraction kit (Intron Biotechnology, Inc., Korea) following the manufacturer instructions. Extracted RNA was stored at -80°C for further analysis.

Maxime one-step RT-PCR premix kit (Intron Biotechnology, Inc., Korea) was used for reverse transcription reaction and amplification of the target region using consensus sense and antisense primers to amplify the capsid pre-membrane (C-PrM) region [27,36]. Template RNA and specific primers were added into the Maxime RT-PCR premix tubes as follows: 5 μl RNA template, 10 pmol forward primer DC1: (5’-TCAATATGCTGAAACGCGCGAGAAACCG-3’), 10 pmol reverse primer DC2: (5’-TTGCACCAACAGTCAATGTCTTCAGGTTC-3’), then the volume was adjusted up to 25 μl by adding 17 μl of nuclease-free water. Positive and negative controls were included. The RT-PCR cycling was performed by reverse transcription at 50°C for 40 min, denaturation at 95°C for 2 min followed by 35 cycles of denaturation (94°C for 30 seconds), primer annealing (55°C for 60 seconds), and extension (72°C for 1.0 seconds) in the thermal cycler (Sensoquest, Germany). The final extension step was performed at 72°C for 10 minutes. A 5μl of the PCR product was electrophoresed on a 1.5% agarose gel in 1X TBE buffer (*pH* 8.3) and visualized under UV using the BDA gel documentation system (Biometra Inc., Germany).

### Household questionnaire to assess risk factors

A pre-tested questionnaire was used to collect the demographic, socioeconomic characteristics, and potential exposure risk factors. The demographic characteristics included age, sex, marital status, level of education, occupation, and residence in Kassala state. The collected socioeconomic data included number of income generators and total individuals living in the house, number of children under 5 years living in the house, types of house units, number of bedrooms, construction materials of the roof, wall, and floor in the house, fuel used for lighting and cooking, source of drinking water, types of toilet and bathroom, type of kitchen, method of disposal of solid waste, number and types of trees around the house, air cooling system, types of transportation and communication, and source of health information and awareness. Information on dengue infection history, and prevention and control methods were collected via asking about the management of water containers in the house, presence of insect screens in the windows, use of bed nets, access to health facilities, travel to endemic dengue sites, vaccination of Yellow Fever, having febrile illness during last three months, heard about dengue, source of dengue, had dengue infection before or any other member in the house, and how the transmission of dengue occurs.

### Statistical analysis

Entomological transmission indices were calculated as described by WHO [33]. Spearman correlation analysis was used to assess correlation between vector densities (mature and immature stages) and microclimatic factors. Analysis of variance (ANOVA) was used to compare the vector mean density between different clusters.

Several indicators were combined together to measure socioeconomic status including occupation, house type, construction materials, and household facilities. To obtain the independent risk factors, all the variables were entered in the multiple logistic regression model (considering that all variables are biologically plausible and there are no confounders) to estimate adjusted matched odds ratios and 95% confidence interval (CI). Statistical analysis was performed using statistical packages for social sciences (SPSS ver. 21) and STATA software (STATA version, StataCorp LP).

## Results

### Demographic and socioeconomic characteristics

A total of 409 households were surveyed during this study, including 254 (62%) in Khatmia, 80 (19.6%) in West Gash, 49 (12%) in Thoriba, and 26 (6.4%) in Shokriya. Majority of the participants were in the 20–39 years’ age group (n = 186, 45.5%) (P<0.01), and with an equal gender distribution except in West Gash cluster (n = 55, 68.8%) (S1 File).

The number of individuals living in the same house in Kassala state ranged between 6 and 10 (n = 237, 57.9%) (S1 File) with secondary school as predominant level of education (n = 119, 29.1%) (P<0.01) (S1 File). About one-third of respondents (n = 140, 34.2%) across all clusters were not formally employed: 42.1% in Khatmia (n = 107), 30.0% in West Gash (n = 24) whereas in Thoriba and Shokriya, majority of study participants were homemakers (n = 6, 23.1%) or free workers (n = 13, 26.5%) (S1 File). A total of 343 respondents (83.9%) from all clusters claimed that they were residents in Kassala state for more than 10 years, (P<0.05) with most of them (n = 406, 99.8%) having lived for their whole life in Kassala state in all clusters (S1 File). Most households in all clusters were semidetached (n = 408, 99.8%) and most participants had a low level of socioeconomic status (n = 269, 65.8%) (P<0.001) (S1 File).

The source of drinking water in most houses, was tap/piped water (n = 403, 98.5%) P<0.01) and nearly half of the water containers were covered with a proper lid (n = 231, 59.4%) and located indoors (n = 67, 17.2%) (P<0.001) except in West Gash (n = 41, 51.3%), and in Shokrya (n = 13, 50%) where most containers were not covered with proper lid (S1 File).

Most respondents used the shared flush toilet (n = 263, 64.9%) (P<0.001) between clusters, except in Shokryia where uncovered pit latrine was common (n = 25, 96.2%) (S1 File). A high percentage (n = 311, 76.6%) of the houses in all clusters had a bathroom built inside their houses (P<0.001), except in West Gash (n = 48, 60.8%;) and Thoriba (n = 28, 57.1%) clusters where the bathroom located outside their houses (S1 File). The majority of respondents kept solid waste in bins (n = 363, 93.6%) (S1 File).

Use of water sprinkles evaporation (n = 92, 24.7%) (P<0.001) was the most common air-cooling system except in West Gash where wood shelter was common (S1 File). Most houses (n = 242, 62.4%) did not have their own transportation (P<0.001). However, most of the people (n = 18, 36.7%) had a motor vehicle for transportation in Thoriba (S1 File). Most houses (n = 386, 99.2%) used a mobile phone for their communication (S1 File). Majority of houses (n = 393, 96.1%) were also without insect screens in the windows (n = 393, 96.1%) (S1 File). In most houses (n = 231, 56.5%), residents used bed nets during sleeping times except in West Gash (n = 36, 45%) and in Shokryia (n = 14, 53.8%) where residents did not use bed nets at all (S1 File). Most people (n = 340, 83.1%) had vaccination against yellow fever (P<0.05) (S1 File). About 77.8% of the participants (n = 318) did not report febrile illness during the last three months (S1 File). Most households (n = 228, 55.7%) claimed that they had heard about dengue infection (P<0.001), except in the West Gash cluster where majority (n = 65, 81.3%) had not heard previously about the disease (S1 File). About half of the respondents (n = 119, 52.2%) obtained the dengue information from their neighbours. Nevertheless, mass media (radio, Television, newspaper, etc.) was the primary source of information about dengue for most people (n = 8, 53.3%) in West Gash (S1 File).

Most of the respondents (n = 221, 96.9%) claimed that they had not previous dengue infection (P<0.01) (S1 File). Again, most people in all clusters (n = 217, 95.2%) claimed that none of their family members had dengue infection before (P<0.001) (S1 File). Most houses in all clusters (n = 114, 50.2%) said dengue could be transmitted through mosquito bites (P<0.05). However, most households in Khatmia (n = 67, 44.1%) and West Gash (n = 9, 60%) said they do not know how the transmission of dengue occurs (S1 File). More details of the characteristics of the respondents are presented in the supporting information (S1 File).

### Seroprevalence of DENV infection

Anti-DENV IgG were detected in 66 (42.04%) out of a total of 157 serum samples. The highest seroprevalence of dengue was identified in Khatmia cluster where 75.9% (n = 22) of samples were positive (Table 1).

**Table 1:** Seroprevalence of dengue infections among randomly selected people in studied households in Kassala state, eastern Sudan during 2016–2017 using IgG-ELISA.

Cluster name	Positive	Total
Khatmia	22 (75.9%)	29
Shokryia	7 (53.85%)	13
Thoriba	20 (42.62%)	42
West Ghash	17 (23.29%)	73
Total	66 (42.04%)	157

### Morphological identification and entomological indices of DENV vector

A total number of 3,304 and 390 *Ae*. *aegypti* larvae and pupae were identified in all study clusters, respectively. The overall house index (HI) was 406 (32.8%) (P<0.01) (S2 File). The HI of containers in all clusters was 57.1% (4/7); however, the total HI for all vessels was 17% (146/857) (S3 File). The overall container index (CI) varied among clusters and ranged from 10.3% in Shokryia to 21.5% in Thoriba (S4 File). The total Breteau Index (BI) was 35.96% (146/406) (S5 File). The overall pupal demographic index (P/D) was 13.31%, and the pupal children index (P/C) was 97.26% (S6 File).

More than 12% (81/627) of containers were covered, 33% (32/97) were partially covered and 24.8% (33/133) were uncovered containers. All these container types were positive for aquatic stages of dengue vector (S7 File). Details of position and breeding status of all container types among the clusters are found in the supporting information (S8 File). The Mean density of *Ae*. *aegypti* (unfed and blood-fed adults) according to different clusters is summarized in Table 2.

**Table 2:** Mean density number of adult *Aedes aegypti* (unfed and blood-fed) according to different clusters in Kassala state, 2016–2017.

	Cluster	No. of houses	Mean ± Std. D.	Std. Error	Kruskal-Wallis Test
**No. of fed mosquitoes**	Khatmia	222	0.07 ± 0.32	0.02	P > 0.05
	Shokryia	18	0.00 ± 0.00	0.00	
	Thoriba	31	0.10 ± 0.40	0.07	
	West Ghash	55	0.02 ± 0.13	0.02	
**No. of unfed mosquitoes**	Khatmia	222	0.19 ±0.48	0.03	P < 0.05
	Shokryia	18	0.00 ± 0.00	0.00	
	Thoriba	31	0.16 ± 0.52	0.09	
	West Ghash	55	0.04 ± 0.27	0.04	
**Number of adults**	Khatmia	222	0.26 ± 0.63	0.04	P < 0.05
	Shokryia	18	0.00 ± 0.00	0.00	
	Thoriba	31	0.26 ± 0.73	0.13	
	West Ghash	55	0.05 ± 0.40	0.05	

The correlation coefficient of temperature and relative humidity with immature stages of *Ae*. *aegypti* in all clusters was -0.022 and -0.05, respectively indicating a poor correlation. While the correlation coefficient of temperature, relative humidity, number of fed mosquitoes, and number of unfed mosquitoes in all clusters was -0.193 (P = 0.001), 0.151 (P = 0.008), 0.576 (P = 0.000) and 0.902 (P = 0.000), respectively.

### Detection of DENV using reverse transcriptase RT-PCR

A total of 187 buffy coat samples were collected from households in all study sites. The 511 bp band of the DENV capsid pre-membrane (C-PrM) region gene was successfully amplified in 2 samples (1.07%) from the Khatmia cluster (Table 3).

**Table 3:** Results of reverse transcriptase PCR of dengue infection in buffy coat and *Aedes aegypti* mosquitoes in studied households in Kassala state, 2016–2017.

Cluster code	Positive buffy coat sample	Total	Positive *Ae*. *aegypti*	Total
Khatmia	2 (1.07%)	187	1 (0.3%)	226
Shokryia	0 (0%)	0	0	19
Thoriba	0 (0%)	0	0	29
West Ghash	0 (0%)	0	0	55
Total	2 (1.07%)	187	1 (0.3%)	329

A total of 329 *Ae*. *aegypti* adults were analyzed for the detection of DENV using the RT-PCR technique. Viral RNA was detected in one out of 329 *Ae*. *aegypti* adults (0.3%) in the Khatmia cluster showing the 511 bp band of the dengue virus (C-PrM) region gene (Table 3). Distribution of DENV positive and negative cases and *Ae*. *aegypti* vectors (immature and adult stages) is presented in S9 File.

### Risk factors analysis

Four variables were identified as independent risk factors in the multivariate analysis (Table 4). These variables included (i) older participants (> 60 years) (OR = 6.31, CI = 1.09–36.36), (ii) type of bathroom in the house (OR = 3.52, CI = 1.35–9.20), (iii) using water-based air conditioner in the house (OR = 6.90, CI = 1.78–26.85), and (iv) any member in the house who had dengue infection before (OR = 28.73, CI = 3.31–249.63).

**Table 4:** Multivariate analysis using multiple logistic regression model for the study variables and dengue infections among the population studied in Kassala state, 2016–2017.

Variable	Odds Ratio	Standard Error	P-value	95% Confidence Interval	
**Age**					
20–39 years	4.20	3.36	0.07	0.88	20.12
40–60 years	2.09	1.75	0.38	0.40	10.85
> 60 years	6.31	5.64	0.04*	1.09	36.36
**Sex**	0.73	0.29	0.43	0.33	1.59
**No. of individuals living in the house**					
6–10 persons	0.96	0.43	0.93	0.40	2.30
> 10 persons	0.14	0.15	0.06	0.02	1.11
Staying in Kassala state	1.31	0.85	0.67	0.37	4.66
**No. of children under 5 years living in the house**					
1–3 child	0.87	0.36	0.74	0.39	1.97
**Roof constructed materials of the house**					
Iron sheets	0.85	0.83	0.87	0.13	5.73
Grass	0.78	1.27	0.88	0.03	18.93
**Wall constructed materials of the house**					
Bricks with mud	0.74	0.49	0.65	0.20	2.71
Cement blocks	0.53	0.45	0.46	0.10	2.84
**Floor constructed materials of the house**					
Cement screed	1.02	0.94	0.98	0.17	6.18
Mud/Sand	1.00	0.98	1.00	0.15	6.78
**Socioeconomic level**					
Medium	11.39	14.17	0.05	0.99	130.57
Low	10.49	20.27	0.22	0.24	463.17
**Management of water containers**	1.52	0.64	0.33	0.66	3.49
**Type of toilet used in the house**	0.47	0.26	0.17	0.16	1.38
**Type of bathroom used in the house**	3.52	1.72	0.01*	1.35	9.20
**Solid waste disposal method**					
Bin-trash	0.23	0.29	0.25	0.02	2.85
Heap	1.10	1.70	0.95	0.05	22.97
**Type of kitchen**	1.70	0.98	0.36	0.55	5.26
**Trees at the house**	0.66	0.25	0.26	0.31	1.37
**Air-cooling system**					
Water-based air conditioner	6.90	4.78	0.01*	1.78	26.85
**Screen in the windows**	0.25	0.27	0.19	0.03	2.04
**Using bed net**	1.84	0.73	0.12	0.85	4.01
**Travelling to Red Sea state during last 3 months**	1.44	0.96	0.59	0.39	5.31
**Yellow fever vaccination**	1.96	0.98	0.18	0.73	5.24
**Having febrile illness during last 3 months**	1.03	0.51	0.96	0.39	2.73
**Heard about dengue**	3.68	4.58	0.30	0.32	42.32
**Had dengue before**	3.72	4.32	0.26	0.38	36.23
**Any household had dengue before**	28.73	31.69	0.00*	3.31	249.63
**Transmission of dengue**					
Don’t know	1.36	0.78	0.59	0.44	4.20

(*) Statistical significant

## Discussion

Seroprevalence, entomological, and socioeconomic community surveys were conducted in four randomly selected endemic sites of dengue in Kassala state, eastern Sudan during 2016–2017. Seroprevalence of the dengue virus has been estimated in this study to be 42%. This was higher than the previous estimated seroprevalence of dengue in Kassala state which was only 9.4% [37]. The increased seroprevalence may be influenced by active circulation and repeated outbreaks of dengue infections in Kassala state [26]. Many studies have reported the seroprevalence of dengue using diverse serological assays in diverse endemic sites, for example, haemagglutination inhibition in Venezuela [38], ELISA in Vietnam and Sudan [39,40]. Other tests used in previous studies in Sudan include RAPID-cassette test in Port Sudan [41], ELISA in Kassala [28,29,31], ELISA and real-time PCR in Kassala [42], ELISA in Gedarif [43]. It is well documented that assessing of seropositivity of dengue cases using IgG is suitable for investigating DENV IgG antibodies levels among people living in endemic sites where repeated outbreaks [44] such as the condition in the current study.

Vector surveillance is considered as a quantifiable routine practice to measure fluctuations of dengue vector populations in endemic countries [45]. Morphological identification showed the dominance of *Ae*. *aegypti* in the study areas. It was reported that this species is found in urbanized areas, and many abiotic factors including temperature, humidity, and rainfall are strongly associated with its abundance [46]. Only one (0.3%) *Ae*. *aegypti* was found positive for Dengue infection in the Khatmia cluster. The latter finding reflects that virus detection in natural populations of mosquitoes is usually challenging, costly, and time-consuming [45].

Larvae and pupae of *Aedes* vector in the present study have been collected from domestic containers in houses as described previously [46]. Items made of pottery were key productive containers for aquatic stages of dengue vector similar to previous studies done in Kassala and Gedarif states [47] and Port Sudan [48]. *Ae*. *albopictus* has not been found during this study, although this species has much broader distribution than its limited native range in Southeast Asia, islands of Indian Ocean, and the western Pacific [49]. Recently, *Ae*. *albopictus* caused public threats by expanding its range to Africa, Europe, and the Americas via human activities and active transportations [3]. Indeed, there is no published report about the presence of *Ae*. *albopictus* in eastern part of Sudan [3].

Vector indices such as HI, CI, and BI are key risk factors and potential early warning indicators for dengue epidemics [50]. Nevertheless, the relationship between these indices and dengue infection is not direct [51], because dengue transmission is complex and dynamic, varying through time and space [45]. The overall HI in all clusters was 32.8%, and this result is comparable with 19% reported in Kassala in 2007 [47]. The HI in Kassala was lower than in other endemic areas, such as in Kinondoni, Tanzania (35.5%) [52], and southern Thailand (60% - 77%) [53]. The BI in the current study (35.96%) was lower than the previously reported BI in Kassala (39.6%) [47], southern Thailand (200%) [53] but higher than 22.2% in Shmal Aldalta locality, Sudan [47]. The CI was varied between areas and ranged between 10.3% and 21.5%. This finding is similar to the CI calculated from Kassala locality in 2007 [47], and lower than those reported in Tanzania [52] and in southern Thailand [53]. The total P/D and P/C were calculated in all clusters, and these indices were measured for the first time in Kassala state, as far as is known.

Urbanization, lack of reticulated clean water systems, overcrowding, and poor housing contribute to large dengue epidemics in many tropical countries [54]. In the present study, four potential risk factors have been identified to be associated with dengue infection in the study sites. These factors are elder age (> 60 years), living in a household with an indoor bathroom, using the water-based air conditioner, and previous dengue infection among members of the household. The increased dengue infection risk among the older individuals has also been reported from another recent study in Kassala state [29]. Two previous studies also reported the older age as a risk factor in Kenya and Colombia [55,56]. Good literacy was strongly associated with dengue transmission in addition to the age in Kenya [55]. Unlike our study, another study in Kassala showed strong associations between dengue circulation and lack of knowledge about dengue disease and household density of more than 3 people per room [37]. A recent study in Vietnam showed a strong association between dengue infection and dengue education, the presence of mosquito larvae in old tires containing water, and proximity to a densely inhabited area [39]. Siregar and colleagues [57] found that history of the previous infection of dengue hemorrhagic fever in the family was one of the risk factors, similar to our observations in the present study. Other risk factors included travel history of family members, frequency of garbage disposal and source of drinking water. Interestingly, old and unscreened housings were associated with dengue transmission in Florida, USA [45,57]. In a comprehensive study that reported poverty-related socioeconomic factors and constraints related to intradomiciliary, potential mosquito breeding sites were linked with a greater risk of acquiring a dengue infection [38]. Adam and colleagues in 2010 reported the effect of dengue among pregnant women and their newborn babies in Port Sudan [58]. The use of water-based air conditioners in the household was identified as a risk factor in the present study and it is in agreement with recent studies elsewhere [3,59]. In contrary, a study in China, has reported that using air-condition was an effective personal prevention measure [60].

Various climatic factors such as temperature, rainfall, and relative humidity affect the vector competence and interact with herd immunity, dengue serotype, and genotype on regional and local scales and seem to be important contributing factor to increasing epidemics [61,62]. Surprisingly, the correlation coefficient of temperature, relative humidity, and number of fed and unfed *Ae*. *aegypti* mosquitoes was significant in all clusters in the present study. Recent study in China has demonstrated the great impact of rains, temperature, and precipitation on DENV transmission [63].

Great progress can be achieved in mapping the distribution of vectors and predicting their spread and dynamics using new technologies such as remote sensing, geographical information system (GIS), and mathematical models. These approaches use demographic, social, climatic, and landscape variables to better offer the health service programs [45]. Surprisingly, the present study provided an additional evidence for spatial overlaps of DENV and mosquito vector occurrences in all study sites (S9 File). The development of a climate-driven spatiotemporal prediction model and the use of GIS in dengue surveillance are essential to inform disease prevention and control interventions [45]. Our future research will consider detailed analyses of dengue incidence and prevalence considering diverse climatic and socioeconomic factors to fill the gap in knowledge available for this recent dengue outbreak.

The study marked some limitations, for example, we assessed the previous history of dengue infection in the respondent’s family member. This kind of information is prone to recall bias in response of study participants, particularly if we could not confirm the family member in a serological test. The study might also miss the information of the family member who had mild or asymptomatic dengue infection. However, we believe this is a random error and affected both case and control group equally and thus the recall bias would not change our results substantially. Our future studies should also consider longer surveillance activities that consider more localities and longer times. The latter may include other localities in Kassala state and other neighboring states to provide better understanding of dengue epidemiology in Sudan.

## Conclusions

Successful dengue control programs will consider using seroprevalence, entomological and socioeconomic information to inform dengue transmission, and to engage the community in reducing vector breeding sites. In addition, the presence of a communicable disease center is critical for estimating and managing future disease risks. The seroprevalence and associated risk factors have been identified in the present study in Kassala state, eastern Sudan. Continued vector surveillance is crucial in endemic areas such as Sudan. Regular integration of entomological, social, environmental, and demographic surveillance data should be considered for better understanding of dengue circulation. All these surveillance types require efficient collaboration between vector-control and public health programs, and be made available on a shared, easily accessible platform.

## Supporting information

S1 File Results of socioeconomic and knowledge attitude and practice (KAP) variables in different clusters in Kassala state, eastern Sudan during 2016–2017. (DOCX)

S2 File Results of House Index (HI) in different clusters in Kassala state, eastern Sudan during 2016–2017. (DOCX)

S3 File Results of House Index (HI) per water container in different clusters in Kassala state, eastern Sudan during 2016–2017. (DOCX)

S4 File Results of Container Index (CI) in different clusters in Kassala state, eastern Sudan during 2016–2017. (DOCX)

S5 File Results of Breteau Index (BI) in different clusters in Kassala state, eastern Sudan during 2016–2017. (DOCX)

S6 File Results of Demographic Index (DI) in different clusters in Kassala state, eastern Sudan during 2016–2017. (DOCX)

S7 File Results of positive container according to covering in different clusters in Kassala state, eastern Sudan during 2016–2017. (DOCX)

S8 File Results of positive container according to type and location of container in different clusters in Kassala state, eastern Sudan during 2016–2017. (DOCX)

S9 File Distribution of dengue cases (positive and negative) and *Aedes aegypti* vector (immature and adult stages) among households of the study sites of Khatmia, Shokriya, West Gash and Thoriba in Kassala state, eastern Sudan during 2016–2017. (TIF)
