# Supplementary material for: Seroprevalence and associated risk factors of Dengue fever in Kassala state, eastern Sudan
Source: PLoS Negl Trop Dis. 2020 Dec 9;14(12):e0008918. doi: 10.1371/journal.pntd.0008918 (PMC7752093; doi:10.1371/journal.pntd.0008918)
Supplement: S1 File — (DOCX) [file pntd.0008918.s001.docx]

**S1 File. Results of socio-economic and knowledge attitude and practice (KAP) variables in different clusters in Kassala state, eastern Sudan during 2016 - 2017**

| **Variable** | **Cluster name** | | | | **Total** | **Significance** |
| --- | --- | --- | --- | --- | --- | --- |
|  | **Khatmia** | **Shokriya** | **Thoriba** | **West Gash** |  |  |
| **Age groups** | | | | | | P < 0.01 |
| < 20 | 17 | 4 | 7 | 12 | 40 |  |
|  | 6.7% | 15.4% | 14.3% | 15.0% | 9.8% |  |
| 20 - 39 | 114 | 9 | 15 | 48 | 186 |  |
|  | 44.9% | 34.6% | 30.6% | 60.0% | 45.5% |  |
| 40 - 60 | 70 | 8 | 19 | 18 | 115 |  |
|  | 27.6% | 30.8% | 38.8% | 22.5% | 28.1% |  |
| > 60 | 53 | 5 | 8 | 2 | 68 |  |
|  | 20.9% | 19.2% | 16.3% | 2.5% | 16.6% |  |
| Total | 254 | 26 | 49 | 80 | 409 |  |
|  | 100.0% | 100.0% | 100.0% | 100.0% | 100.0% |  |
| **Sex** | | | | | | P < 0.01 |
| Male | 136 | 16 | 27 | 25 | 204 |  |
|  | 53.5% | 61.5% | 55.1% | 31.3% | 49.9% |  |
| Female | 118 | 10 | 22 | 55 | 205 |  |
|  | 46.5% | 38.5% | 44.9% | 68.8% | 50.1% |  |
| Total | 254 | 26 | 49 | 80 | 409 |  |
|  | 100.0% | 100.0% | 100.0% | 100.0% | 100.0% |  |
| **Number of living individuals in the house** | | | | | | P > 0.01 |
| 0 - 5 | 84 | 11 | 19 | 21 | 135 |  |
|  | 33.1% | 42.3% | 38.8% | 26.3% | 33.0% |  |
| 6 - 10 | 156 | 10 | 27 | 44 | 237 |  |
|  | 61.4% | 38.5% | 55.1% | 55.0% | 57.9% |  |
| > 10 | 14 | 5 | 3 | 15 | 37 |  |
|  | 5.5% | 19.2% | 6.1% | 18.8% | 9.0% |  |
| Total | 254 | 26 | 49 | 80 | 409 |  |
|  | 100.0% | 100.0% | 100.0% | 100.0% | 100.0% |  |
| **Marital status** | | | | | | P > 0.05 |
| Single | 70 | 8 | 17 | 32 | 127 |  |
|  | 27.6% | 30.8% | 34.7% | 40.0% | 31.1% |  |
| Married (Monogamy) | 177 | 17 | 31 | 46 | 271 |  |
|  | 69.7% | 65.4% | 63.3% | 57.5% | 66.3% |  |
| Divorced/separated | 1 | 0 | 0 | 1 | 2 |  |
|  | 0.4% | 0.0% | 0.0% | 1.3% | 0.5% |  |
| Widowed | 6 | 1 | 1 | 1 | 9 |  |
|  | 2.4% | 3.8% | 2.0% | 1.3% | 2.2% |  |
| Total | 254 | 26 | 49 | 80 | 409 |  |
|  | 100.0% | 100.0% | 100.0% | 100.0% | 100.0% |  |

| **Level of education** | | | | | | | | | | P < 0.01 |
| --- | --- | --- | --- | --- | --- | --- | --- | --- | --- | --- |
| Never attended school | 20 | | 5 | | 3 | | 7 | | 35 |  |
|  | 7.9% | | 19.2% | | 6.1% | | 8.8% | | 8.6% |  |
| Informal | 18 | | 3 | | 2 | | 19 | | 42 |  |
|  | 7.1% | | 11.5% | | 4.1% | | 23.8% | | 10.3% |  |
| Primary school | 66 | | 9 | | 13 | | 25 | | 113 |  |
|  | 26.0% | | 34.6% | | 26.5% | | 31.3% | | 27.6% |  |
| Intermediate school | 22 | | 1 | | 4 | | 3 | | 30 |  |
|  | 8.7% | | 3.8% | | 8.2% | | 3.8% | | 7.3% |  |
| Secondary school | 75 | | 6 | | 19 | | 19 | | 119 |  |
|  | 29.5% | | 23.1% | | 38.8% | | 23.8% | | 29.1% |  |
| College/certificate | 52 | | 2 | | 8 | | 7 | | 69 |  |
|  | 20.5% | | 7.7% | | 16.3% | | 8.8% | | 16.9% |  |
| Higher education | 1 | | 0 | | 0 | | 0 | | 1 |  |
|  | .4% | | 0.0% | | 0.0% | | 0.0% | | .2% |  |
| Total | 254 | | 26 | | 49 | | 80 | | 409 |  |
|  | 100.0% | | 100.0% | | 100.0% | | 100.0% | | 100.0% |  |
| **Occupation** | | | | | | | | | |  |
| Student | 1 | | 0 | | 0 | | 0 | | 1 |  |
|  | .4% | | 0.0% | | 0.0% | | 0.0% | | .2% |  |
| Accounter | 2 | | 0 | | 0 | | 0 | | 2 |  |
|  | .8% | | 0.0% | | 0.0% | | 0.0% | | .5% |  |
| Blacksmith | 0 | | 0 | | 0 | | 2 | | 2 |  |
|  | 0.0% | | 0.0% | | 0.0% | | 2.5% | | .5% |  |
| Cleaner | 1 | | 0 | | 0 | | 0 | | 1 |  |
|  | .4% | | 0.0% | | 0.0% | | 0.0% | | .2% |  |
| Doesn't work | 107 | | 3 | | 6 | | 24 | | 140 |  |
|  | 42.1% | | 11.5% | | 12.2% | | 30.0% | | 34.2% |  |
| Driver | 19 | | 0 | | 1 | | 0 | | 20 |  |
|  | 7.5% | | 0.0% | | 2.0% | | 0.0% | | 4.9% |  |
| Farmer | 4 | | 5 | | 0 | | 0 | | 9 |  |
|  | 1.6% | | 19.2% | | 0.0% | | 0.0% | | 2.2% |  |
| Free work | 39 | | 4 | | 13 | | 7 | | 63 |  |
|  | 15.4% | | 15.4% | | 26.5% | | 8.8% | | 15.4% |  |
| Geologist | 1 | | 0 | | 0 | | 0 | | 1 |  |
|  | .4% | | 0.0% | | 0.0% | | 0.0% | | .2% |  |
| Guard | 2 | | 0 | | 0 | | 0 | | 2 |  |
|  | .8% | | 0.0% | | 0.0% | | 0.0% | | .5% |  |
| Homemaker | 1 | | 6 | | 13 | | 22 | | 42 |  |
|  | .4% | | 23.1% | | 26.5% | | 27.5% | | 10.3% |  |
| Industrial | 0 | | 0 | | 1 | | 0 | | 1 |  |
|  | 0.0% | | 0.0% | | 2.0% | | 0.0% | | .2% |  |
| Labor | 2 | | 1 | | 1 | | 0 | | 4 |  |
|  | .8% | | 3.8% | | 2.0% | | 0.0% | | 1.0% |  |
| Laboratories | 0 | | 0 | | 1 | | 0 | | 1 |  |
|  | 0.0% | | 0.0% | | 2.0% | | 0.0% | | .2% |  |
| Lawyer | 0 | | 0 | | 1 | | 0 | | 1 |  |
|  | 0.0% | | 0.0% | | 2.0% | | 0.0% | | .2% |  |
| Leader of a mosque | 2 | | 0 | | 1 | | 0 | | 3 |  |
|  | .8% | | 0.0% | | 2.0% | | 0.0% | | .7% |  |
| Manager Assistant | 1 | | 0 | | 0 | | 0 | | 1 |  |
|  | .4% | | 0.0% | | 0.0% | | 0.0% | | .2% |  |
| Mayor | 1 | | 0 | | 0 | | 0 | | 1 |  |
|  | .4% | | 0.0% | | 0.0% | | 0.0% | | .2% |  |
| Mechanic | 3 | | 0 | | 0 | | 1 | | 4 |  |
|  | 1.2% | | 0.0% | | 0.0% | | 1.3% | | 1.0% |  |
| Nurse | 1 | | 1 | | 0 | | 0 | | 2 |  |
|  | .4% | | 3.8% | | 0.0% | | 0.0% | | .5% |  |
| Officer | 9 | | 0 | | 0 | | 0 | | 9 |  |
|  | 3.5% | | 0.0% | | 0.0% | | 0.0% | | 2.2% |  |
| Policeman | 5 | | 0 | | 1 | | 2 | | 8 |  |
|  | 2.0% | | 0.0% | | 2.0% | | 2.5% | | 2.0% |  |
| Putcher | 1 | | 0 | | 0 | | 0 | | 1 |  |
|  | .4% | | 0.0% | | 0.0% | | 0.0% | | .2% |  |
| Retired | 9 | | 0 | | 2 | | 0 | | 11 |  |
|  | 3.5% | | 0.0% | | 4.1% | | 0.0% | | 2.7% |  |
| Saler | 1 | | 0 | | 0 | | 0 | | 1 |  |
|  | .4% | | 0.0% | | 0.0% | | 0.0% | | .2% |  |
| Solider | 2 | | 0 | | 0 | | 2 | | 4 |  |
|  | .8% | | 0.0% | | 0.0% | | 2.5% | | 1.0% |  |
| Student | 16 | | 5 | | 5 | | 13 | | 39 |  |
|  | 6.3% | | 19.2% | | 10.2% | | 16.3% | | 9.5% |  |
| Tailor | 2 | | 0 | | 0 | | 0 | | 2 |  |
|  | .8% | | 0.0% | | 0.0% | | 0.0% | | .5% |  |
| Teacher | 11 | | 0 | | 1 | | 2 | | 14 |  |
|  | 4.3% | | 0.0% | | 2.0% | | 2.5% | | 3.4% |  |
| Trader | 7 | | 1 | | 2 | | 0 | | 10 |  |
|  | 28.0% | | 3.8% | | 4.1% | | 0.0% | | 2.4% |  |
| Veterinerist | 1 | | 0 | | 0 | | 0 | | 1 |  |
|  | .4% | | 0.0% | | 0.0% | | 0.0% | | .2% |  |
| Worker | 2 | | 0 | | 0 | | 5 | | 7 |  |
|  | .8% | | 0.0% | | 0.0% | | 6.3% | | 1.7% |  |
| Total | 254 | | 26 | | 49 | | 80 | | 409 |  |
|  | 100.0% | | 100.0% | | 100.0% | | 100.0% | | 100.0% |  |
| **Number of working individuals at the house** | | | | | | | | | | P < 0.001 |
| 0 | 49 | 1 | | 2 | | 4 | | 56 | |  |
|  | 19.3% | 3.8% | | 4.1% | | 5.0% | | 13.7% | |  |
| 1 | 169 | 20 | | 23 | | 33 | | 245 | |  |
|  | 66.5% | 76.9% | | 46.9% | | 41.3% | | 59.9% | |  |
| 2 | 35 | 5 | | 12 | | 21 | | 73 | |  |
|  | 13.8% | 19.2% | | 24.5% | | 26.3% | | 17.8% | |  |
| > 2 | 1 | 0 | | 12 | | 22 | | 35 | |  |
|  | .4% | 0.0% | | 24.5% | | 27.5% | | 8.6% | |  |
| Total | 254 | 26 | | 49 | | 80 | | 409 | |  |
|  | 100.0% | 100.0% | | 100.0% | | 100.0% | | 100.0% | |  |
| **Duration of staying at the house** | | | | | | | | | | P < 0.05 |
| All time | 201 | 25 | | 44 | | 73 | | 343 | |  |
|  | 79.1% | 96.2% | | 89.8% | | 91.3% | | 83.9% | |  |
| Not always | 53 | 1 | | 5 | | 7 | | 66 | |  |
|  | 20.9% | 3.8% | | 10.2% | | 8.8% | | 16.1% | |  |
| Total | 254 | 26 | | 49 | | 80 | | 409 | |  |
|  | 100.0% | 100.0% | | 100.0% | | 100.0% | | 100.0% | |  |
| **Continuously living in Kassala state, Sudan** | | | | | | | | | | P > 0.05 |
| Kassala | 252 | 26 | | 49 | | 79 | | 406 | |  |
|  | 99.6% | 100.0% | | 100.0% | | 100.0% | | 99.8% | |  |
| Red Sea state | 1 | 0 | | 0 | | 0 | | 1 | |  |
|  | .4% | 0.0% | | 0.0% | | 0.0% | | .2% | |  |
| Total | 253 | 26 | | 49 | | 79 | | 407 | |  |
|  | 100.0% | 100.0% | | 100.0% | | 100.0% | | 100.0% | |  |
| **Children under 5 years living at the house** | | | | | | | | | | P > 0.05 |
| 0 | 122 | 11 | | 23 | | 39 | | 195 | |  |
|  | 48.0% | 42.3% | | 46.9% | | 48.8% | | 47.4% | |  |
| 1 -3 | 128 | 13 | | 25 | | 36 | | 202 | |  |
|  | 50.4% | 50.0% | | 51.0% | | 45.0% | | 49.4% | |  |
| > 3 | 4 | 2 | | 1 | | 5 | | 12 | |  |
|  | 1.6% | 7.7% | | 2.0% | | 6.3% | | 2.9% | |  |
| Total | 254 | 26 | | 49 | | 80 | | 409 | |  |
|  | 100.0% | 100.0% | | 100.0% | | 100.0% | | 100.0% | |  |
| **Type of housing unit** | | | | | | | | | | P > 0.05 |
| Detached house | 1 | 0 | | 0 | | 0 | | 1 | |  |
|  | .4% | 0.0% | | 0.0% | | 0.0% | | .2% | |  |
| Semidetached house | 253 | 26 | | 49 | | 80 | | 408 | |  |
|  | 99.6% | 100.0% | | 100.0% | | 100.0% | | 99.8% | |  |
| Total | 254 | 26 | | 49 | | 80 | | 409 | |  |
|  | 100.0% | 100.0% | | 100.0% | | 100.0% | | 100.0% | |  |
| **Socioeconomic status** | | | | | | | | | | P < 0.001 |
| Low | 161 | 25 | | 15 | | 68 | | 269 | |  |
|  | 63.4% | 96.2% | | 30.6% | | 85.0% | | 65.8% | |  |
| Medium | 68 | 1 | | 13 | | 11 | | 93 | |  |
|  | 26.8% | 3.8% | | 26.5% | | 13.8% | | 22.7% | |  |
| High | 25 | 0 | | 21 | | 1 | | 47 | |  |
|  | 9.8% | 0.0% | | 42.9% | | 1.3% | | 11.5% | |  |
| Total | 254 | 26 | | 49 | | 80 | | 409 | |  |
|  | 100.0% | 100.0% | | 100.0% | | 100.0% | | 100.0% | |  |
| **Source of drinking water at the house** | | | | | | | | | | P < 0.01 |
| Tap/piped water | 253 | 26 | | 49 | | 75 | | 403 | |  |
|  | 99.6% | 100.0% | | 100.0% | | 93.8% | | 98.5% | |  |
| Water truck/water vendor | 1 | 0 | | 0 | | 5 | | 6 | |  |
|  | .4% | 0.0% | | 0.0% | | 6.3% | | 1.5% | |  |
| Total | 254 | 26 | | 49 | | 80 | | 409 | |  |
|  | 100.0% | 100.0% | | 100.0% | | 100.0% | | 100.0% | |  |
| **Water container management and location at the house** | | | | | | | | | | P < 0.001 |
| Covered with proper lid | 144 | 11 | | 37 | | 39 | | 231 | |  |
|  | 61.5% | 42.3% | | 75.5% | | 48.8% | | 59.4% | |  |
| Uncovered with proper lid | 32 | 13 | | 4 | | 41 | | 90 | |  |
|  | 13.7% | 50.0% | | 8.2% | | 51.3% | | 23.1% | |  |
| Found outdoor | 0 | 0 | | 1 | | 0 | | 1 | |  |
|  | 0.0% | 0.0% | | 2.0% | | 0.0% | | .3% | |  |
| Found indoor | 58 | 2 | | 7 | | 0 | | 67 | |  |
|  | 24.8% | 7.7% | | 14.3% | | 0.0% | | 17.2% | |  |
| Total | 234 | 26 | | 49 | | 80 | | 389 | |  |
|  | 100.0% | 100.0% | | 100.0% | | 100.0% | | 100.0% | |  |
| **Type of toilet in the house** | | | | | | | | | | P < 0.001 |
| Covered pit latrine (private) | 19 | 0 | | 0 | | 0 | | 19 | |  |
|  | 7.6% | 0.0% | | 0.0% | | 0.0% | | 4.7% | |  |
| Covered pit latrine (shared) | 5 | 0 | | 0 | | 1 | | 6 | |  |
|  | 2.0% | 0.0% | | 0.0% | | 1.3% | | 1.5% | |  |
| Uncovered pit latrine | 25 | 25 | | 0 | | 34 | | 84 | |  |
|  | 10.0% | 96.2% | | 0.0% | | 42.5% | | 20.7% | |  |
| Flush toilet (private) | 32 | 0 | | 0 | | 1 | | 33 | |  |
|  | 12.8% | 0.0% | | 0.0% | | 1.3% | | 8.1% | |  |
| Flush toilet (shared) | 169 | 1 | | 49 | | 44 | | 263 | |  |
|  | 67.6% | 3.8% | | 100.0% | | 55.0% | | 64.9% | |  |
| Total | 250 | 26 | | 49 | | 80 | | 405 | |  |
|  | 100.0% | 100.0% | | 100.0% | | 100.0% | | 100.0% | |  |
| **Location of bathroom in the house** | | | | | | | | | | P < 0.001 |
| Inside | 239 | 20 | | 21 | | 31 | | 311 | |  |
|  | 94.8% | 76.9% | | 42.9% | | 39.2% | | 76.6% | |  |
| Outside (built) | 13 | 6 | | 28 | | 48 | | 95 | |  |
|  | 5.2% | 23.1% | | 57.1% | | 60.8% | | 23.4% | |  |
| Total | 252 | 26 | | 49 | | 79 | | 406 | |  |
|  | 100.0% | 100.0% | | 100.0% | | 100.0% | | 100.0% | |  |
| **Disposable of solid waste at the house** | | | | | | | | | | P > 0.05 |
| Bin-trash | 210 | 26 | | 49 | | 78 | | 363 | |  |
|  | 89.7% | 100.0% | | 100.0% | | 98.7% | | 93.6% | |  |
| Pit | 1 | 0 | | 0 | | 0 | | 1 | |  |
|  | .4% | 0.0% | | 0.0% | | 0.0% | | .3% | |  |
| Heap | 15 | 0 | | 0 | | 0 | | 15 | |  |
|  | 6.4% | 0.0% | | 0.0% | | 0.0% | | 3.9% | |  |
| Burning | 8 | 0 | | 0 | | 1 | | 9 | |  |
|  | 3.4% | 0.0% | | 0.0% | | 1.3% | | 2.3% | |  |
| Total | 234 | 26 | | 49 | | 79 | | 388 | |  |
|  | 100.0% | 100.0% | | 100.0% | | 100.0% | | 100.0% | |  |
| **Location of kitchen in the house** | | | | | | | | | | P < 0.001 |
| Inside | 251 | 26 | | 41 | | 29 | | 347 | |  |
|  | 98.8% | 100.0% | | 83.7% | | 36.3% | | 84.8% | |  |
| Outside (built) | 3 | 0 | | 8 | | 50 | | 61 | |  |
|  | 1.2% | 0.0% | | 16.3% | | 62.5% | | 14.9% | |  |
| None | 0 | 0 | | 0 | | 1 | | 1 | |  |
|  | 0.0% | 0.0% | | 0.0% | | 1.3% | | .2% | |  |
| Total | 254 | 26 | | 49 | | 80 | | 409 | |  |
|  | 100.0% | 100.0% | | 100.0% | | 100.0% | | 100.0% | |  |
| **Number of trees and bushes at house** | | | | | | | | | | P > 0.05 |
| None | 116 | 11 | | 15 | | 27 | | 169 | |  |
|  | 45.7% | 42.3% | | 30.6% | | 33.8% | | 41.3% | |  |
| 1 -3 | 124 | 15 | | 32 | | 52 | | 223 | |  |
|  | 48.8% | 57.7% | | 65.3% | | 65.0% | | 54.5% | |  |
| > 3 | 14 | 0 | | 2 | | 1 | | 17 | |  |
|  | 5.5% | 0.0% | | 4.1% | | 1.3% | | 4.2% | |  |
| Total | 254 | 26 | | 49 | | 80 | | 409 | |  |
|  | 100.0% | 100.0% | | 100.0% | | 100.0% | | 100.0% | |  |
| **Air cooling system in the house** | | | | | | | | | | P < 0.001 |
| Sprinkle water | 86 | 0 | | 2 | | 4 | | 92 | |  |
|  | 39.4% | 0.0% | | 4.1% | | 5.0% | | 24.7% | |  |
| Iron sheet shelter | 41 | 1 | | 11 | | 25 | | 78 | |  |
|  | 18.8% | 3.8% | | 22.4% | | 31.3% | | 20.9% | |  |
| Grasses shelter | 44 | 21 | | 7 | | 15 | | 87 | |  |
|  | 20.2% | 80.8% | | 14.3% | | 18.8% | | 23.3% | |  |
| Wood shelter | 26 | 3 | | 2 | | 36 | | 67 | |  |
|  | 11.9% | 11.5% | | 4.1% | | 45.0% | | 18.0% | |  |
| Water-based air conditioner | 15 | 1 | | 27 | | 0 | | 43 | |  |
|  | 6.9% | 3.8% | | 55.1% | | 0.0% | | 11.5% | |  |
| Split unit | 2 | 0 | | 0 | | 0 | | 2 | |  |
|  | .9% | 0.0% | | 0.0% | | 0.0% | | .5% | |  |
| Others | 4 | 0 | | 0 | | 0 | | 4 | |  |
|  | 1.8% | 0.0% | | 0.0% | | 0.0% | | 1.1% | |  |
| Total | 218 | 26 | | 49 | | 80 | | 373 | |  |
|  | 100.0% | 100.0% | | 100.0% | | 100.0% | | 100.0% | |  |
| **Type of transportation used in the house** | | | | | | | | | | P < 0.001 |
| Motor vehicle | 38 | 3 | | 18 | | 1 | | 60 | |  |
|  | 16.3% | 11.5% | | 36.7% | | 1.3% | | 15.5% | |  |
| Motor cycle | 8 | 0 | | 7 | | 19 | | 34 | |  |
|  | 3.4% | 0.0% | | 14.3% | | 23.8% | | 8.8% | |  |
| Bicycle | 13 | 0 | | 9 | | 26 | | 48 | |  |
|  | 5.6% | 0.0% | | 18.4% | | 32.5% | | 12.4% | |  |
| Donkey/camel | 1 | 0 | | 0 | | 3 | | 4 | |  |
|  | .4% | 0.0% | | 0.0% | | 3.8% | | 1.0% | |  |
| None | 173 | 23 | | 15 | | 31 | | 242 | |  |
|  | 74.2% | 88.5% | | 30.6% | | 38.8% | | 62.4% | |  |
| Total | 233 | 26 | | 49 | | 80 | | 388 | |  |
|  | 100.0% | 100.0% | | 100.0% | | 100.0% | | 100.0% | |  |
| **Type of communication used at the house** | | | | | | | | | | P > 0.05 |
| Television | 1 | 0 | | 0 | | 0 | | 1 | |  |
|  | .4% | 0.0% | | 0.0% | | 0.0% | | .3% | |  |
| Mobile phone | 233 | 26 | | 48 | | 79 | | 386 | |  |
|  | 99.6% | 100.0% | | 98.0% | | 98.8% | | 99.2% | |  |
| Fixed phone | 0 | 0 | | 1 | | 1 | | 2 | |  |
|  | 0.0% | 0.0% | | 2.0% | | 1.3% | | .5% | |  |
| Total | 234 | 26 | | 49 | | 80 | | 389 | |  |
|  | 100.0% | 100.0% | | 100.0% | | 100.0% | | 100.0% | |  |
| **Source of information in the house** | | | | | | | | | | P > 0.05 |
| Electronic media | 16 | 0 | | 0 | | 0 | | 16 | |  |
|  | 6.8% | 0.0% | | 0.0% | | 0.0% | | 4.1% | |  |
| Words of mouth | 218 | 26 | | 49 | | 80 | | 373 | |  |
|  | 92.8% | 100.0% | | 100.0% | | 100.0% | | 95.6% | |  |
| Others | 1 | 0 | | 0 | | 0 | | 1 | |  |
|  | .4% | 0.0% | | 0.0% | | 0.0% | | .3% | |  |
| Total | 235 | 26 | | 49 | | 80 | | 390 | |  |
|  | 100.0% | 100.0% | | 100.0% | | 100.0% | | 100.0% | |  |
| **Screens in windows at the house** | | | | | | | | | | P > 0.05 |
| Screens present and intact | 5 | 1 | | 1 | | 2 | | 8 | |  |
|  | 2.0% | 3.8% | | 2.0% | | 2.6% | | 2.2% | |  |
| Screens present and not intact | 5 | 0 | | 0 | | 1 | | 6 | |  |
|  | 2.0% | 0.0% | | 2.0% | | 1.3% | | 1.7% | |  |
| No screens | 244 | 25 | | 47 | | 77 | | 393 | |  |
|  | 96.1% | 96.2% | | 95.9% | | 96.3% | | 96.1% | |  |
| Total | 254 | 26 | | 49 | | 80 | | 409 | |  |
|  | 100.0% | 100.0% | | 100.0% | | 100.0% | | 100.0% | |  |
| **Use of bed-nets in the house** | | | | | | | | | | P > 0.05 |
| Use it daily | 167 | 5 | | 30 | | 29 | | 231 | |  |
|  | 65.7% | 19.2% | | 61.2% | | 36.3% | | 56.5% | |  |
| Use it occasionally | 53 | 7 | | 7 | | 15 | | 82 | |  |
|  | 20.9% | 26.9% | | 14.3% | | 18.8% | | 20.0% | |  |
| Don’t use | 34 | 14 | | 12 | | 36 | | 96 | |  |
|  | 13.4% | 53.8% | | 24.5% | | 45.0% | | 23.5% | |  |
| Total | 254 | 26 | | 49 | | 80 | | 409 | |  |
|  | 100.0% | 100.0% | | 100.0% | | 100.0% | | 100.0% | |  |
| **Travel to Red Sea state during the last three months** | | | | | | | | | | P > 0.05 |
| Yes | 19 | 0 | | 3 | | 10 | | 32 | |  |
|  | 7.5% | 0.0% | | 6.1% | | 12.5% | | 7.8% | |  |
| No | 235 | 26 | | 46 | | 70 | | 377 | |  |
|  | 92.5% | 100.0% | | 93.9% | | 87.5% | | 92.2% | |  |
| Total | 254 | 26 | | 49 | | 80 | | 409 | |  |
|  | 100.0% | 100.0% | | 100.0% | | 100.0% | | 100.0% | |  |
| **Any household member travelled to Red Sea state in the last three months** | | | | | | | | | | P > 0.05 |
| Yes | 29 | 0 | | 11 | | 13 | | 53 | |  |
|  | 11.4% | 0.0% | | 22.4% | | 16.3% | | 13.0% | |  |
| No | 224 | 26 | | 38 | | 67 | | 355 | |  |
|  | 88.2% | 100.0% | | 77.6% | | 83.8% | | 86.8% | |  |
| Decline | 1 | 0 | | 0 | | 0 | | 1 | |  |
|  | .4% | 0.0% | | 0.0% | | 0.0% | | .2% | |  |
| Total | 254 | 26 | | 49 | | 80 | | 409 | |  |
|  | 100.0% | 100.0% | | 100.0% | | 100.0% | | 100.0% | |  |
| **Vaccination of Yellow fever** | | | | | | | | | | P < 0.05 |
| Yes | 216 | 25 | | 39 | | 60 | | 340 | |  |
|  | 85.0% | 96.2% | | 79.6% | | 75.0% | | 83.1% | |  |
| No | 35 | 0 | | 10 | | 20 | | 65 | |  |
|  | 13.8% | 0.0% | | 20.4% | | 25.0% | | 15.9% | |  |
| Decline | 3 | 1 | | 0 | | 0 | | 4 | |  |
|  | 1.2% | 3.8% | | 0.0% | | 0.0% | | 1.0% | |  |
| Total | 254 | 26 | | 49 | | 80 | | 409 | |  |
|  | 100.0% | 100.0% | | 100.0% | | 100.0% | | 100.0% | |  |
| **Having febrile illness in the last three months** | | | | | | | | | | P > 0.05 |
| Yes | 54 | 9 | | 7 | | 15 | | 85 | |  |
|  | 21.3% | 34.6% | | 14.3% | | 18.8% | | 20.8% | |  |
| No | 195 | 17 | | 42 | | 64 | | 318 | |  |
|  | 76.8% | 65.4% | | 85.7% | | 80.0% | | 77.8% | |  |
| Don’t remember | 5 | 0 | | 0 | | 1 | | 6 | |  |
|  | 2.0% | 0.0% | | 0.0% | | 1.3% | | 1.5% | |  |
| Total | 254 | 26 | | 49 | | 80 | | 409 | |  |
|  | 100.0% | 100.0% | | 100.0% | | 100.0% | | 100.0% | |  |
| **Heard about Dengue** | | | | | | | | | | P < 0.001 |
| Yes | 153 | 24 | | 36 | | 15 | | 228 | |  |
|  | 60.2% | 92.3% | | 73.5% | | 18.8% | | 55.7% | |  |
| No | 101 | 2 | | 13 | | 65 | | 181 | |  |
|  | 39.8% | 7.7% | | 26.5% | | 81.3% | | 44.3% | |  |
| Total | 254 | 26 | | 49 | | 80 | | 409 | |  |
|  | 100.0% | 100.0% | | 100.0% | | 100.0% | | 100.0% | |  |
| **Source of the information about dengue** | | | | | | | | | | P. > 0.05 |
| Home visitors | 1 | 0 | | 0 | | 0 | | 1 | |  |
|  | .7% | 0.0% | | 0.0% | | 0.0% | | .4% | |  |
| Mass media (radio,TV, newspaper) | 30 | 0 | | 2 | | 8 | | 40 | |  |
|  | 19.6% | 0.0% | | 5.6% | | 53.3% | | 17.5% | |  |
| Medical/health cadre | 9 | 7 | | 3 | | 2 | | 21 | |  |
|  | 5.9% | 29.2% | | 8.3% | | 13.3% | | 9.2% | |  |
| Mobile microphone | 8 | 0 | | 1 | | 2 | | 11 | |  |
|  | 5.2% | 0.0% | | 2.8% | | 13.3% | | 4.8% | |  |
| Beer/family member | 17 | 1 | | 3 | | 1 | | 22 | |  |
|  | 11.1% | 4.2% | | 8.3% | | 6.7% | | 9.6% | |  |
| Neighbors | 85 | 16 | | 18 | | 0 | | 119 | |  |
|  | 55.6% | 66.7% | | 50.0% | | 0.0% | | 52.2% | |  |
| Reading | 0 | 0 | | 0 | | 2 | | 2 | |  |
|  | 0.0% | 0.0% | | 0.0% | | 13.3% | | .9% | |  |
| Total | 153 | 24 | | 36 | | 15 | | 228 | |  |
|  | 100.0% | 100.0% | | 100.0% | | 100.0% | | 100.0% | |  |
| **Had dengue infection before** | | | | | | | | | | P < 0.01 |
| Yes | 1 | 3 | | 3 | | 0 | | 7 | |  |
|  | .7% | 12.5% | | 8.3% | | 0.0% | | 3.1% | |  |
| No | 152 | 21 | | 33 | | 15 | | 221 | |  |
|  | 99.3% | 87.5% | | 91.7% | | 100.0% | | 96.9% | |  |
| Total | 153 | 24 | | 36 | | 15 | | 228 | |  |
|  | 100.0% | 100.0% | | 100.0% | | 100.0% | | 100.0% | |  |
| **Any household member had dengue infection before** | | | | | | | | | | P < 0.001 |
| Yes | 1 | 7 | | 3 | | 0 | | 11 | |  |
|  | .7% | 29.2% | | 8.3% | | 0.0% | | 4.8% | |  |
| No | 152 | 17 | | 33 | | 15 | | 217 | |  |
|  | 99.3% | 70.8% | | 91.7% | | 100.0% | | 95.2% | |  |
| Total | 153 | 24 | | 36 | | 15 | | 228 | |  |
|  | 100.0% | 100.0% | | 100.0% | | 100.0% | | 100.0% | |  |
| **Dengue transmission** | | | | | | | | | | P < 0.05 |
| Mosquito bite | 64 | 18 | | 26 | | 6 | | 114 | |  |
|  | 42.1% | 75.0% | | 72.2% | | 40.0% | | 50.2% | |  |
| Drinking contaminated water | 1 | 1 | | 0 | | 0 | | 2 | |  |
|  | .7% | 4.2% | | 0.0% | | 0.0% | | .9% | |  |
| Eating contaminated food | 1 | 0 | | 0 | | 0 | | 1 | |  |
|  | .7% | 0.0% | | 0.0% | | 0.0% | | .4% | |  |
| Direct contact with infected person | 1 | 0 | | 0 | | 0 | | 1 | |  |
|  | .7% | 0.0% | | 0.0% | | 0.0% | | .4% | |  |
| Don’t know | 67 | 5 | | 9 | | 9 | | 90 | |  |
|  | 44.1% | 20.8% | | 25.0% | | 60.0% | | 39.6% | |  |
| Others | 18 | 0 | | 1 | | 0 | | 19 | |  |
|  | 11.8% | 0.0% | | 2.8% | | 0.0% | | 8.4% | |  |
| Total | 152 | 24 | | 36 | | 15 | | 227 | |  |
|  | 100.0% | 100.0% | | 100.0% | | 100.0% | | 100.0% | |  |
