# Supplementary material for: Seroprevalence and associated risk factors of Dengue fever in Kassala state, eastern Sudan
Source: PLoS Negl Trop Dis. 2020 Dec 9;14(12):e0008918. doi: 10.1371/journal.pntd.0008918 (PMC7752093; doi:10.1371/journal.pntd.0008918)
Supplement: S2 File — (DOCX) [file pntd.0008918.s002.docx]

**S2 File. Results of House Index (HI) in different clusters in Kassala state, eastern Sudan during 2016 – 2017.**

| Cluster name | House Index (HI) Total House Number |
| --- | --- |
| Khatmia | (27.3%) 253 |
| Shokryia | (26.9%) 26 |
| Thoriba | (40.4%) 47 |
| West Ghash | (47.5%) 80 |
| All study area | (32.8%) 406 |
