# Supplementary material for: Seroprevalence and associated risk factors of Dengue fever in Kassala state, eastern Sudan
Source: PLoS Negl Trop Dis. 2020 Dec 9;14(12):e0008918. doi: 10.1371/journal.pntd.0008918 (PMC7752093; doi:10.1371/journal.pntd.0008918)
Supplement: S3 File — (DOCX) [file pntd.0008918.s003.docx]

**S3 File. Results of House Index (HI) per water container in different clusters in Kassala state, eastern Sudan during 2016 – 2017.**

| Container | Index |
| --- | --- |
| Water-based air conditioner | 57.10% (4/7) |
| Iron bucket | 12.50% (2/16) |
| Plastic collector | 0.00% (0/8) |
| Pottery | 15.40% (82/534) |
| Water basin | 85.70% (6/7) |
| Water container | 18.30% (52/284) |
| Water tank | 0.00% (0/1) |
| Total | 17.00% (146/857) |
