# Supplementary material for: Seroprevalence and associated risk factors of Dengue fever in Kassala state, eastern Sudan
Source: PLoS Negl Trop Dis. 2020 Dec 9;14(12):e0008918. doi: 10.1371/journal.pntd.0008918 (PMC7752093; doi:10.1371/journal.pntd.0008918)
Supplement: S4 File — (DOCX) [file pntd.0008918.s004.docx]

**S4 File. Results of Container Index (CI) in different clusters in Kassala state, eastern Sudan during 2016 – 2017.**

| Container | Clusters | | | |
| --- | --- | --- | --- | --- |
|  | **Khatmia** | **Shokryia** | **Thoriba** | **West Ghash** |
| Iron bucket | 12.50% (2/16) | - | - | - |
| Plastic collector | 0.00% (0/8) | - | - | - |
| Pottery | 20.60% (43/209) | 3.40% (2/58) | 4.30% (2/46) | 15.80% (35/221) |
| Water basin | 100.0% (2/2) | 100% (4/4) | 0.00% (0/1) | - |
| Water container | 23.30% (28/120) | 11.40% (4/35) | 32.10% (17/53) | 3.90% (3/76) |
| Water tank | 0.00% (0/1) | - | - | - |
| Water-based air conditioner | - | - | 57.10% (4/7) | - |
| Total | 21.10% (75/356) | 10.30% (10/97) | 21.50% (23/107) | 12.80% (38/297) |
