# Supplementary material for: Seroprevalence and associated risk factors of Dengue fever in Kassala state, eastern Sudan
Source: PLoS Negl Trop Dis. 2020 Dec 9;14(12):e0008918. doi: 10.1371/journal.pntd.0008918 (PMC7752093; doi:10.1371/journal.pntd.0008918)
Supplement: S5 File — (DOCX) [file pntd.0008918.s005.docx]

**S5 File. Results of Breteau Index (BI) in different clusters in Kassala state, eastern Sudan during 2016 – 2017.**

| Cluster name | Positive | No. of houses | Breteau Index (BI) |
| --- | --- | --- | --- |
| Khatmia | 75 | 253 | 29.64% |
| Shokryia | 10 | 26 | 38.46% |
| Thoriba | 23 | 47 | 48.94% |
| West Ghash | 38 | 80 | 47.50% |
| Total | 146 | 406 | 35.96% |
