# Supplementary material for: Seroprevalence and associated risk factors of Dengue fever in Kassala state, eastern Sudan
Source: PLoS Negl Trop Dis. 2020 Dec 9;14(12):e0008918. doi: 10.1371/journal.pntd.0008918 (PMC7752093; doi:10.1371/journal.pntd.0008918)
Supplement: S6 File — (DOCX) [file pntd.0008918.s006.docx]

**S6 File. Results of Demographic Index (DI) in different clusters in Kassala state, eastern Sudan during 2016 – 2017.**

| Cluster | Larvae | No. pupae | Immature | No. of living individuals | No. of living children | Larvae/  demographic | Larvae/ children | Pupae/  demographic | Pupae/ children | Immature/  demographic | Immature/  children |
| --- | --- | --- | --- | --- | --- | --- | --- | --- | --- | --- | --- |
| Khatmia | 2996 | 365 | 3311 | 1758 | 235 | 170.42% | 1274.89% | 20.76% | 155.32% | 188.34% | 1408.94% |
| Shokryia | 48 | 0 | 48 | 177 | 37 | 27.12% | 129.73% | 0.00% | 0.00% | 27.12% | 129.73% |
| Thoriba | 20 | 5 | 25 | 313 | 44 | 6.39% | 45.45% | 1.60% | 11.36% | 7.99% | 56.82% |
| West Ghash | 240 | 20 | 260 | 683 | 85 | 35.14% | 282.35% | 2.93% | 23.53% | 38.07% | 305.88% |
| Total | 3304 | 390 | 3644 | 2931 | 401 | 112.73% | 823.94% | 13.31% | 97.26% | 124.33% | 908.73% |
