# Supplementary material for: Seroprevalence and associated risk factors of Dengue fever in Kassala state, eastern Sudan
Source: PLoS Negl Trop Dis. 2020 Dec 9;14(12):e0008918. doi: 10.1371/journal.pntd.0008918 (PMC7752093; doi:10.1371/journal.pntd.0008918)
Supplement: S7 File — (DOCX) [file pntd.0008918.s007.docx]

**S7 File. Results of positive container according to covering in different clusters in Kassala state, eastern Sudan during 2016 – 2017.**

| Container status | Positive container | Total Number | % |
| --- | --- | --- | --- |
| Covered | 81 | 627 | 12.90% |
| Partially covered | 32 | 97 | 33.00% |
| Uncovered | 33 | 133 | 24.80% |
| Total | 146 | 857 | 17.00% |
