# Supplementary material for: Seroprevalence and associated risk factors of Dengue fever in Kassala state, eastern Sudan
Source: PLoS Negl Trop Dis. 2020 Dec 9;14(12):e0008918. doi: 10.1371/journal.pntd.0008918 (PMC7752093; doi:10.1371/journal.pntd.0008918)
Supplement: S8 File — (DOCX) [file pntd.0008918.s008.docx]

**S8 File. Results of positive container according to type and location of container in different clusters in Kassala state, eastern Sudan during 2016 – 2017.**

| **Cluster** | **Container** | **Location** | **Breeding** | **Count** | **Total N %** |
| --- | --- | --- | --- | --- | --- |
| Khatmia | Iron bucket | indoor | negative | 1 | 100.00% |
|  |  | outdoor | negative | 13 | 86.70% |
|  |  |  | positive | 2 | 13.30% |
|  | Plastic collector | indoor | negative | 2 | 100.00% |
|  |  | outdoor | negative | 6 | 100.00% |
|  | Pottery | indoor | negative | 51 | 78.50% |
|  |  |  | positive | 14 | 21.50% |
|  |  | outdoor | negative | 115 | 79.90% |
|  |  |  | positive | 29 | 20.10% |
|  | Water basin | indoor | positive | 1 | 100.00% |
|  |  | outdoor | positive | 1 | 100.00% |
|  | Water container | indoor | negative | 47 | 74.60% |
|  |  |  | positive | 16 | 25.40% |
|  |  | outdoor | negative | 45 | 78.90% |
|  |  |  | positive | 12 | 21.10% |
|  | Water tank | outdoor | negative | 1 | 100.00% |
| Shokryia | Pottery | indoor | negative | 23 | 100.00% |
|  |  | outdoor | negative | 33 | 94.30% |
|  |  |  | positive | 2 | 5.70% |
|  | Water basin | outdoor | positive | 4 | 100.00% |
|  | Water container | indoor | negative | 9 | 75.00% |
|  |  |  | positive | 3 | 25.00% |
|  |  | outdoor | negative | 22 | 95.70% |
|  |  |  | positive | 1 | 4.30% |
| Thoriba | Water-based air conditioner | outdoor | negative | 3 | 42.90% |
|  |  |  | positive | 4 | 57.10% |
|  | Pottery | indoor | negative | 6 | 100.00% |
|  |  | outdoor | negative | 38 | 95.00% |
|  |  |  | positive | 2 | 5.00% |
|  | Water basin | outdoor | negative | 1 | 100.00% |
|  | Water container | indoor | negative | 4 | 66.70% |
|  |  |  | positive | 2 | 33.30% |
|  |  | outdoor | negative | 32 | 68.10% |
|  |  |  | positive | 15 | 31.90% |
| West Ghash | Pottery | indoor | negative | 83 | 85.60% |
|  |  |  | positive | 14 | 14.40% |
|  |  | outdoor | negative | 103 | 83.10% |
|  |  |  | positive | 21 | 16.90% |
|  | Water container | indoor | negative | 13 | 100.00% |
|  |  | outdoor | negative | 60 | 95.20% |
|  |  |  | positive | 3 | 4.80% |
