# Supplementary figures and images for: Seroprevalence and associated risk factors of Dengue fever in Kassala state, eastern Sudan
Source: PLoS Negl Trop Dis. 2020 Dec 9;14(12):e0008918. doi: 10.1371/journal.pntd.0008918 (PMC7752093; doi:10.1371/journal.pntd.0008918)

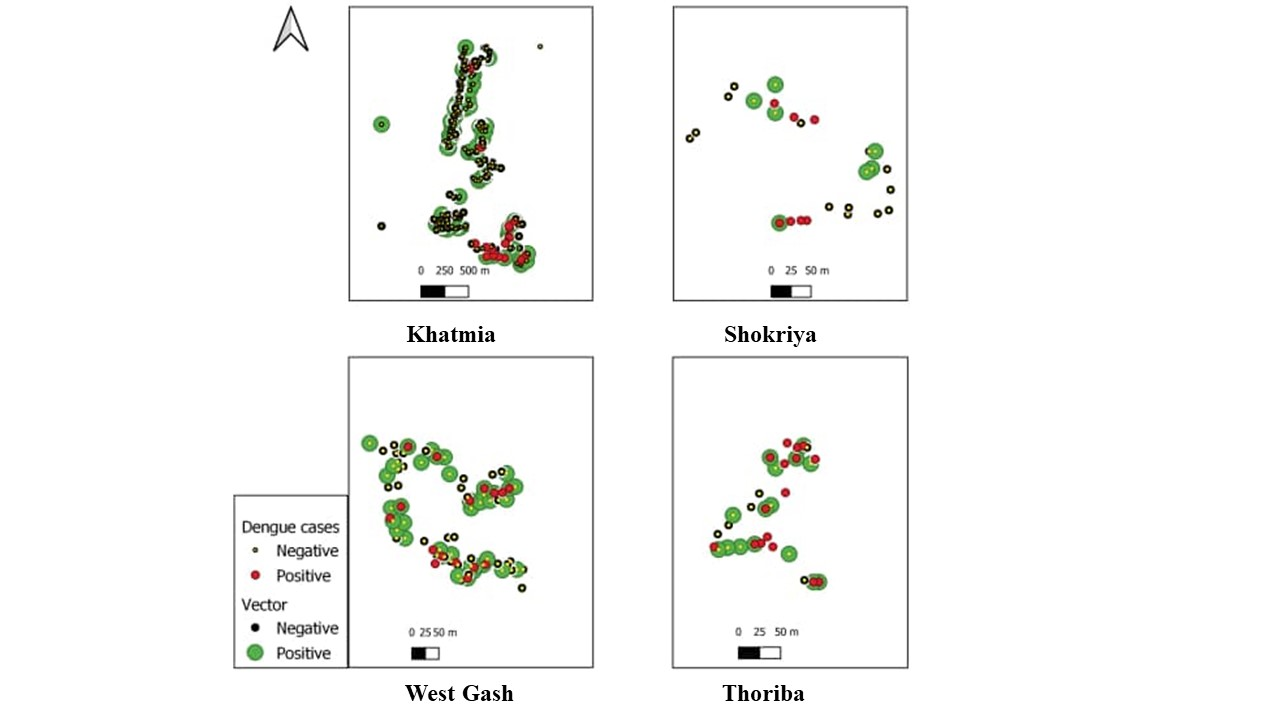

Supplement: S9 File — (TIF) [file pntd.0008918.s009.tif]
